# 

**Published:** 1884-03

**Authors:** 


### CONTENTS.

ORIGINAL COMMUNICATIONS—

Extreme Anæsthesia for Obstetric Operations. By J. McF. Gaston, M.D., Atlanta, Ga. . . . . . . . . . . . . . . . . . . . 1

What of Bismuth? By H. V. M. Miller, M.D., Atlanta, Ga. . . . 5

An Interesting Case of Abortion. By K. P. Moore, M.D., Macon, Georgia . . . . . . . . . . . . . . . . . . . . . . . . 12

Unexpected Result in a Case of Strangulated Hernia. By Geo. R. West, M.D., Rome, Ga. . . . . . . . . . . . . . . . . 15

The Dry Treatment of Suppurative Inflammation of the Middle Ear. By A. W. Calhoun, M.D., Atlanta, Ga. . . . . . . . . 17

SELECTIONS—

Modus Operandi of Zymotic Agents. By W. W. Hipolite, M.D., De Valls Bluff, Ark. . . . . . . . . . . . . . . . . . . . 20

Notes on the Use of Ether in Obstetrics. By Casey Albert Wood, C.M., M.D. . . . . . . . . . . . . . . . . . . . . . . . 31

Panaritium. By Theodor Billroth . . . . . . . . . . . . . . 35

The Danger of Large Doses of Quinine. By A. A. Smith, M.D. . 38

EDITORIAL—

Our Journal . . . . . . . . . . . . . . . . . . . . . . . . 42

Distribution of Scholarships . . . . . . . . . . . . . . . 45

New and Old Code of Ethics in the State of New York . . . . 46

Dr. J. McF. Gaston . . . . . . . . . . . . . . . . . . . . 47

Our Title Page . . . . . . . . . . . . . . . . . . . . . . 48

The Late Dr. J. Marion Sims, . . . . . . . . . . . . . . . 50

Atlanta Medical College . . . . . . . . . . . . . . . . . 53

Our Advertisers . . . . . . . . . . . . . . . . . . . . . . 54

NOTES AND COMMENTS . . . . . . . . . . . . . . 4, 19, 30, 44, 56–64

